# Effect of arginine on oligomerization and stability of N-acetylglutamate synthase

**DOI:** 10.1038/srep38711

**Published:** 2016-12-09

**Authors:** N. Haskins, A. Mumo, P. H. Brown, M. Tuchman, H. Morizono, L. Caldovic

**Affiliations:** 1 Center for Genetic Medicine Research; Children’s National Medical Center, Washington, DC, USA; 2 American Academy of Otolaryngology – Head and Neck Surgery Foundation, Alexandria VA, USA; 3 National Institute of General Medical Sciences, National Institutes of Health, Bethesda, MD, USA

## Abstract

N-acetylglutamate synthase (NAGS; E.C.2.3.1.1) catalyzes the formation of N-acetylglutamate (NAG) from acetyl coenzyme A and glutamate. In microorganisms and plants, NAG is the first intermediate of the L-arginine biosynthesis; in animals, NAG is an allosteric activator of carbamylphosphate synthetase I and III. In some bacteria bifunctional N-acetylglutamate synthase-kinase (NAGS-K) catalyzes the first two steps of L-arginine biosynthesis. L-arginine inhibits NAGS in bacteria, fungi, and plants and activates NAGS in mammals. L-arginine increased thermal stability of the NAGS-K from *Maricaulis maris* (MmNAGS-K) while it destabilized the NAGS-K from *Xanthomonas campestris* (XcNAGS-K). Analytical gel chromatography and ultracentrifugation indicated tetrameric structure of the MmMNAGS-K in the presence and absence of L-arginine and a tetramer-octamer equilibrium that shifted towards tetramers upon binding of L-arginine for the XcNAGS-K. Analytical gel chromatography of mouse NAGS (mNAGS) indicated either different oligomerization states that are in moderate to slow exchange with each other or deviation from the spherical shape of the mNAGS protein. The partition coefficient of the mNAGS increased in the presence of L-arginine suggesting smaller hydrodynamic radius due to change in either conformation or oligomerization. Different effects of L-arginine on oligomerization of NAGS may have implications for efforts to determine the three-dimensional structure of mammalian NAGS.

N-acetylglutamate synthase (NAGS; EC 2.3.1.1) catalyzes the formation of N-acetylglutamate (NAG) from glutamate and acetyl coenzyme A (AcCoA)^1^. In microorganisms and plants, NAG is the first committed intermediate of arginine biosynthesis pathway, whereas in animals, NAG is an allosteric activator of urea cycle enzymes carbamylphosphate synthetase I (CPS1; EC 6.3.4.16) and carbamylphosphate synthetase III (CPS3)^1^,^2^,^3^,^4^,^5^. In mammals, the urea cycle converts ammonia, a waste product of protein catabolism and a potent neurotoxin, into urea, which is easily excreted. Therefore, the physiological role of urea cycle in mammals is to protect their brains from the toxic effects of ammonia. The ability to convert ammonia into non-toxic metabolites is considered a vital evolutionary adaptation that allowed animals to move from water to land^6^.

Fungal and animal NAGS proteins are mitochondrial enzymes^7^,^8^,^9^. Alignments of either mammalian or fish NAGS protein sequences revealed three regions of conservation: the mitochondrial targeting signal, the variable segment and the conserved segment^10^,^11^. Removal of the mitochondrial targeting signal, which is presumably cleaved off upon import into mitochondria, results in the mature NAGS (NAGS-M) whereas conserved NAGS (NAGS-C) lacks both the mitochondrial targeting signal and the variable segment. The function of the variable segment is unknown. The recombinant mouse NAGS-C (mNAGS-C) and zebrafish NAGS-C, which lack the variable segment, have two-fold higher enzymatic activity than the corresponding mature recombinant proteins with the variable segment^10^,^11^. Phylogenetic analysis of NAGS from different organisms revealed two families of NAGS proteins: a classical, or *E. coli*-like, NAGS from most bacteria and plants, and a vertebrate-like NAGS from some bacteria, fungi and animals^12^. The NAGS polypeptide consists of two conserved structural domains, an amino acid kinase domain that harbors the binding site for the allosteric regulator L-arginine and an N-acetyltransferase domain that binds substrates and contains the catalytic site^5^,^12^,^13^,^14^,^15^,^16^,^17^.

L-arginine is an allosteric regulator of NAGS. The binding site for L-arginine is conserved in bacterial, plant and animal NAGS but the allosteric effect of L-arginine on these NAGS proteins differs^6^,^18^. Inhibition of NAGS by L-arginine provides feedback regulation of arginine biosynthesis in microbes and plants^5^,^19^ while in vertebrates with urea cycle L-arginine either partially inhibits or activates NAGS^6^,^11^,^20^,^21^,^22^,^23^. This inversion of the allosteric effect of L-arginine on NAGS enzymatic activity occurred in amphibians^6^. The mechanism of allosteric regulation is different in classical, *E. coli*-like NAGS, and the bifunctional NAGS-K, which are more similar to mammalian NAGS^13^,^24^. Structures of liganded and unliganded NAGS from *Neisseria gonorrhoeae* (NgNAGS), which is *E. coli*-like, revealed that L-arginine inhibits this protein by inducing conformational change, which includes localized disorder of the glutamate binding site^13^. L-arginine inhibits vertebrate-like NAGS from *Maricaulis maris* (MmNAGS-K) by blocking binding of AcCoA^24^. The mechanism of NAGS allosteric regulation by L-arginine may be different in mammalian and vertebrate-like bacterial enzymes because of the different effect of L-arginine on their activity.

L-arginine also appears to affect the oligomerization state of some NAGS proteins. Partially purified NAGS from *E. coli* (EcNAGS) protein is an ensemble of rapidly exchanging oligomers in the absence of L-arginine while in the presence of L-arginine EcNAGS is a hexamer^25^. However, the *E. coli*-like NAGS from *Neisseria gonorrhoeae* (NgNAGS) is a hexamer both in the absence and presence of L-arginine^13^,^16^. The crystal structure of the vertebrate-like NAGS-K from *Xanthomonas campestris* (XcNAGS-K) revealed a tetrameric oligomerization state in the absence of L-arginine^15^; vertebrate-like NAGS-K from *Maricaulis maris* (MmNAGS-K) and *Saccharomyces cerevisiae* N-acetylglutamate kinase, which are highly similar to vertebrate NAGS^12^ are tetramers, both with, and without, L-arginine^15^,^24^,^26^. Unlike its bacterial counterparts, purified rat NAGS appears to change oligomerization state in the presence of L-arginine^9^. Similarly, the oligomerization state of recombinant NAGS from zebrafish (*Danio rerio*) changes in the presence of L-arginine^11^ suggesting that the change in oligomerization state may play a role in allosteric regulation of that NAGS.

Although the three dimensional structures of two bacterial NAGS and a fungal NAGK have been determined^15^,^24^,^26^, the structure of mammalian NAGS remains elusive, possibly because they exist as an ensemble of different oligomers in solution. Therefore, we compared the effect of L-arginine on the oligomerization state and stability of mouse NAGS and bacterial homologs with known three-dimensional structures.

## Results and Discussion

### Effect of L-Arginine on Thermal Unfolding of NAGS

L-arginine is an allosteric regulator of NAGS in microbes, plants and mammals^5^,^6^,^27^. Because the binding site for L-arginine is conserved in all NAGS enzymes^6^,^18^ but the mechanism of allosteric regulation differs in classical and vertebrate-like bacterial enzymes^13^,^24^ we used Thermofluor methodology to determine the effect of the binding of L-arginine on the stability of bacterial and mammalian NAGS.

**Figure 1:**
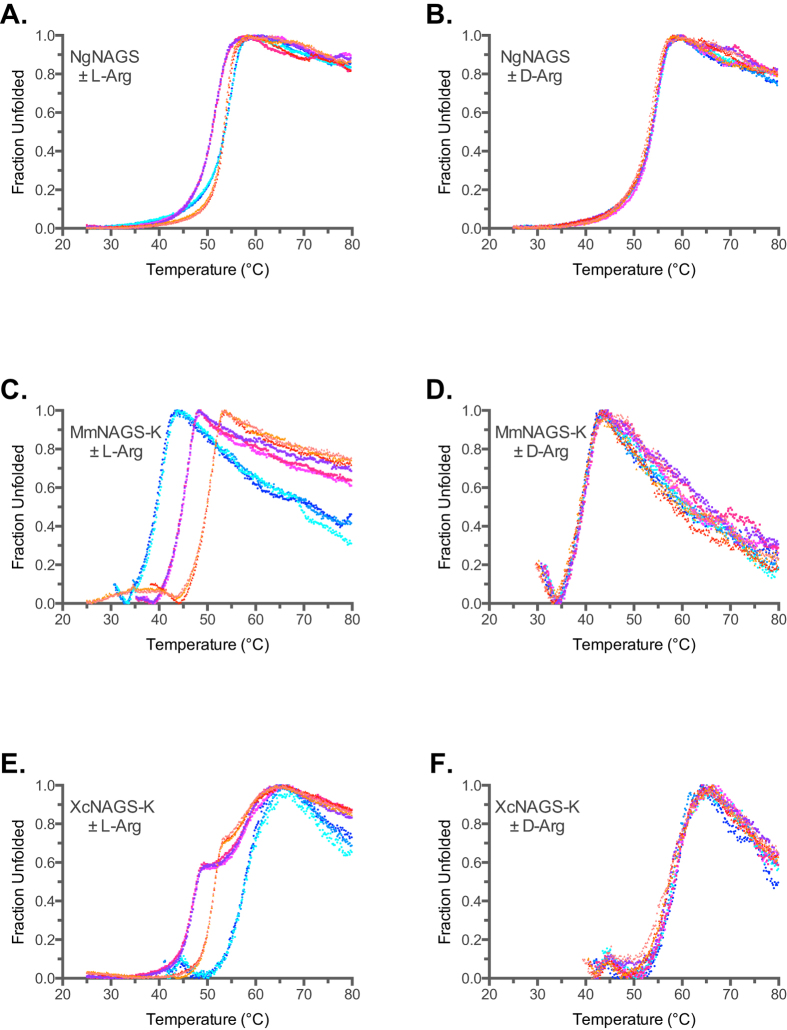
Thermofluor analysis of NgNAGS, MmNAGS-K and XcNAGS-K in the presence and absence of L-arginine and D-arginine. Unfolding of bacterial NgNAGS was measured in the presence of increasing concentrations of either L-arginine (**A**) or D-arginine (**B**). Unfolding of MmNAGS-K was measured in the presence of increasing concentrations of either L-arginine (**C**) or D-arginine (**D**). Unfolding of XcNAGS-K was measured in the presence of increasing concentrations of either L-arginine (**E**) or D-arginine (**F**). Cyan and blue – thermal unfolding in the absence of L- or D-arginine. Magenta - thermal unfolding in the presence of 1 mM L- or D-arginine. Orange - thermal unfolding in the presence of 10 mM L- or D-arginine.

The effects of L-arginine on thermal unfolding of *E. coli*-like NgNAGS and vertebrate-like MmNAGS-K and XcNAGS-K are markedly different (Fig. 1). In the presence of 1 mM L-arginine, NgNAGS appears to be destabilized by approximately 2.5 °C, whereas in the presence of 10 mM L-arginine, the Tm of NgNAGS appears unchanged (Fig. 1A and Table 1). Addition of D-arginine as a negative control did not affect thermal stability of NgNAGS (Fig. 1B). The apparent destabilization of NgNAGS in the presence of 1 mM L-arginine, when NgNAGS monomers and L-arginine are equimolar, could result from the combination of stabilization upon binding of L-arginine and increased disorder of the NAT domain^13^. More likely, NgNAGS hexamer may be partially saturated in the presence of 1 mM L-arginine resulting in less stable, asymetric hexamer with some of its NAT-domains existing in the active, ordered conformation and others in the inactive, disordered conformation. In the presence of 10 mM L-arginine NgNAGS is saturated, with all of its NAT-domains in the inactive conformation resulting in the symmetrical hexamer that is equally stable as the fully active enzyme without L-arginine.

MmNAGS-K was the least stable of proteins examined in this study. In the absence of L-arginine or with D-arginine the Tm of MmNAGS-K was 39.3 ± 0.2 °C (Fig. 1C and D, Table 1; MmNAGS-K is 52% identical to XcNAGS-K). Addition of L-arginine increased the thermal stability of MmNAGS-K. Its Tm increased by approximately 5 °C and 10 °C in the presence of equimolar (1 mM) amount and ten-fold (10 mM) excess of L-arginine, respectively (Fig. 1C and Table 1). The thermal unfolding of XcNAGS-K, which is 52% identical to MmNAGS-K (Supplementary Fig. S1), was distinctly different from the behavior of MmNAGS-K. In the absence of L-arginine or with D-arginine the Tm of XcNAGS-K was approximately 58 °C (Fig. 1E and F, Table 1). In the presence of L-arginine, XcNAGS appears to undergo a multi-state unfolding over a broader temperature interval (Fig. 1E), which may indicate ensembles of molecules with different oligomerization states and Tm values. The lower Tm of the XcNAGS-K in the presence of L-arginine may indicate a quaternary structure with fewer monomers than in the absence of L-arginine.

**Figure 2:**
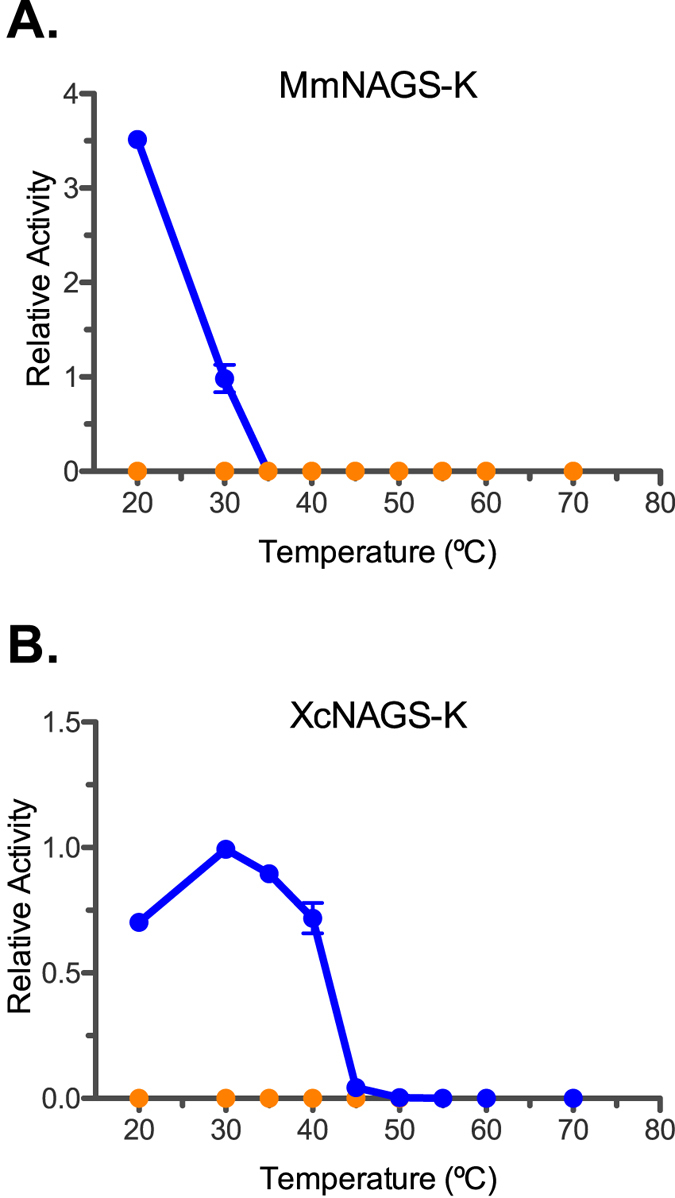
Thermal inactivation of MmNAGS-K (**A**) and XcNAGS-K (**B**). Specific synthase activity was normalized to specific activity measured after incubation at 30 °C. Blue – relative synthase activity in the absence of L-arginine. Orange – relative synthase activity in the presence of 1 mM L-arginine.

The synthase activities of both MmNAGS-K and XcNAGS-K decreased as temperature increased (Fig. 2). Specific activity of MmNAGS-K decreased between 20 °C and 35 °C, while specific activity of XcNAGS-K increased between 20 °C and 30 °C, followed by a decrease as temperature raised to 50 °C. Specific activity of XcNAGS-K decreased at higher temperatures than MmNAGS-K, consistent with the difference in stability of the two proteins in the thermal unfolding assays (Fig. 1). Because both MmNAGS-K and XcNAGS-K are completely inhibited by L-arginine^12^,^15^ synthase activity of both enzymes was zero after incubation at temperatures between 20 °C and 70 °C in the presence of 1 mM L-arginine (Fig. 2).

**Figure 3:**
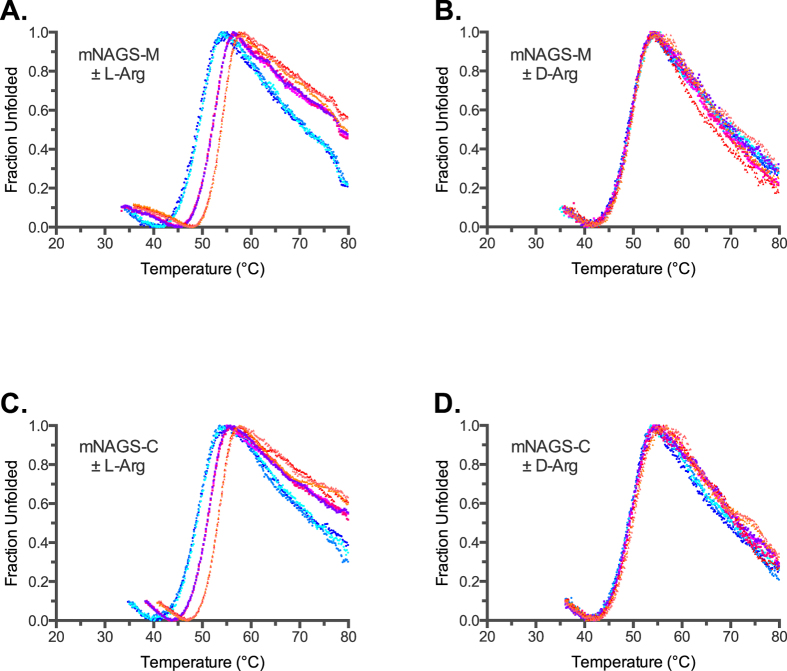
Thermofluor analysis of mouse NAGS in the presence and absence of L- and D-arginine. Unfolding of mNAGS-M was measured in the presence of increasing concentrations of either L-arginine (**A**) or D-arginine (**B**). Unfolding of mNAGS-C was measured in the presence of increasing concentrations of either L-arginine (**C**) or D-arginine (**D**). Cyan and blue – thermal unfolding in the absence of L- or D-arginine. Magenta - thermal unfolding in the presence of 1 mM L- or D-arginine. Orange - thermal unfolding in the presence of 10 mM L- or D-arginine.

**Figure 4:**
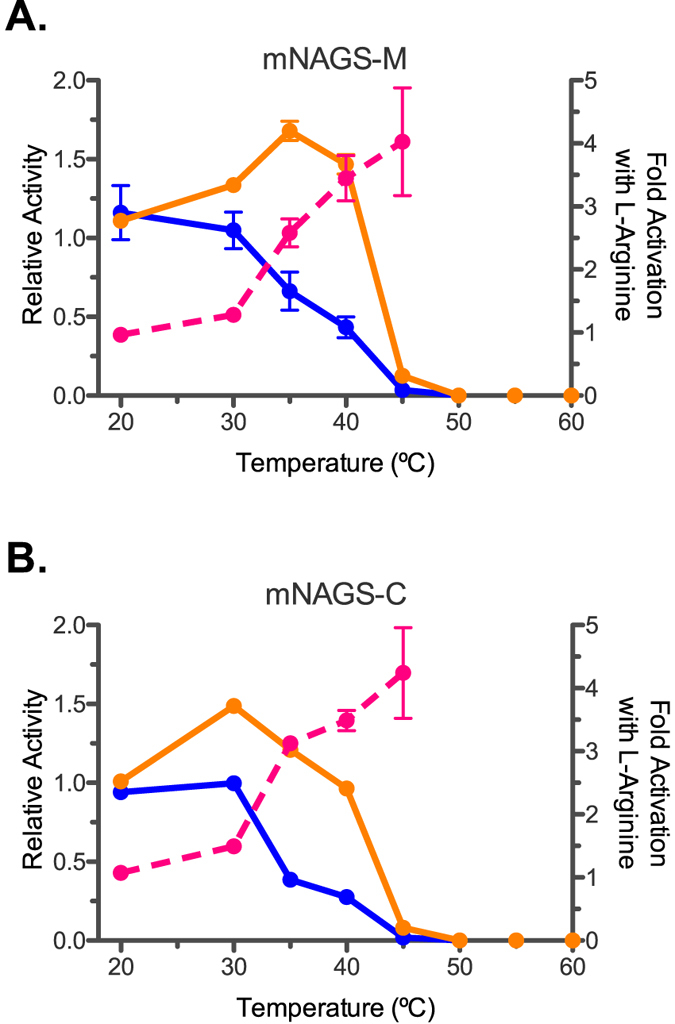
Thermal inactivation of mNAGS-M (**A**) and mNAGS-C (**B**). Specific NAGS activity was normalized to specific activity measured after incubation at 30 °C. Blue – relative synthase activity in the absence of L-arginine after incubation without L-arginine. Orange – relative synthase activity in the presence of 1 mM L-arginine after incubation with 1 mM L-arginine. Magenta – fold-increase of specific NAGS activity in the presence of 1 mM L-arginine.

Both mNAGS-M and mNAGS-C had similar unfolding behaviors (Fig. 3 and Table 1). The Tm of mNAGS-M and mNAGS-C were similar both in the absence of arginine or in the presence of D-arginine (Fig. 3). Both proteins were stabilized by approximately 3 °C and 5 °C in the presence of 1 mM and 10 mM L-arginine, respectively (Fig. 3 and Table 1). Specific activity of both mNAGS-M and mNAGS-C decreased between 30 °C and 50 °C (Fig. 4). In the presence of L-arginine, specific activity of both mNAGS-M and mNAGS-C increased 1.5- to 4-fold between 30 °C and 45 °C (Fig. 4). The increase in activation of both mNAGS-M and mNAGS-C when they were incubated and assayed with 1 mM L-arginine suggests a protective effect of L-arginine on mouse NAGS (Fig. 4). Increased activation of mNAGS-M and mNAGS-C by L-arginine at 30–45 °C most likely reflects change of quaternary and/or tertiary structure, as well as dependence of the equilibrium between two states on temperature and presence of L-arginine. At 50 °C and above both enzymes were completely inactivated regardless of the presence of L-arginine (Fig. 4).

Enzymatic activities of MmNAGS-K, XcNAGS-K, mNAGS-M and mNAGS-C decreased within temperature intervals that were each lower than temperature ranges where each protein unfolded (Figs 1C, 1E, 2, 3A, 3C and 4). This behavior of the four proteins in this study is similar to other proteins that became inactive at temperatures that were lower than temperatures at which circular dicroism indicated unfolding^28^,^29^,^30^,^31^.

The behavior of mNAGS-C was distinctly different from the corresponding zebrafish NAGS-C, which unfolded over a broad temperature interval and was markedly less stable than zebrafish NAGS-M^11^. The mNAGS-C and zebrafish NAGS-C are 49% identical (Supplementary Fig. S1) and both proteins have higher specific activity than mNAGS-M and zebrafish NAGS-M^11^,^21^ but mouse NAGS is activated while zebrafish NAGS is inhibited by the L-arginine^6^,^11^. Therefore, binding of L-arginine is likely to trigger different conformational changes in the mouse and zebrafish NAGS, which may explain their differences in thermal unfolding.

### Hydrodynamic Radii and Molecular Weight of NAGS

Earlier studies of NAGS from bacteria, fish and mammals revealed that the oligomeric state of some of these proteins changes upon binding of L-arginine^11^,^25^,^32^, while others do not^13^,^15^,^16^,^24^. Because the three-dimensional structure of NgNAGS, whose oligomerization state does not change upon binding of L-arginine, is known^13^,^16^, while the structure of NAGS from *E. coli*, whose oligomerization state does change in the presence of L-arginine^25^ remains elusive, we tested if a similar correlation holds for vertebrate-like MmNAGS-K and XcNAGS-K, both with known three-dimensional structures, though the resolution of XcNAGS-M crystals is lower than the MmNAGS-K crystals^15^. We used small-zone analytical gel chromatography to determine if oligomerization states of MmNAGS-K, XcNAGS-K, mNAGS-M and mNAGS-C depend on the concentration of each protein and/or change in the presence of L-arginine.

The elution volumes of MmNAGS-K and XcNAGS-K corresponded to approximate molecular weights of 263 and 277 kDa, respectively (Supplementary Fig. S2). These molecular weights deviate from the calculated molecular weights of MmNAGS-K and XcNAGS-K tetramers (200.5 kDa and 205.9 kDa, respectively). Because crystal structures of MmNAGS-K and XcNAGS-K indicate a tetrameric state for both proteins^15^, this discrepancy could be either due to deviations of their shape from a sphere, or because both proteins are ensembles of molecules with different oligomerization states.

**Figure 5:**
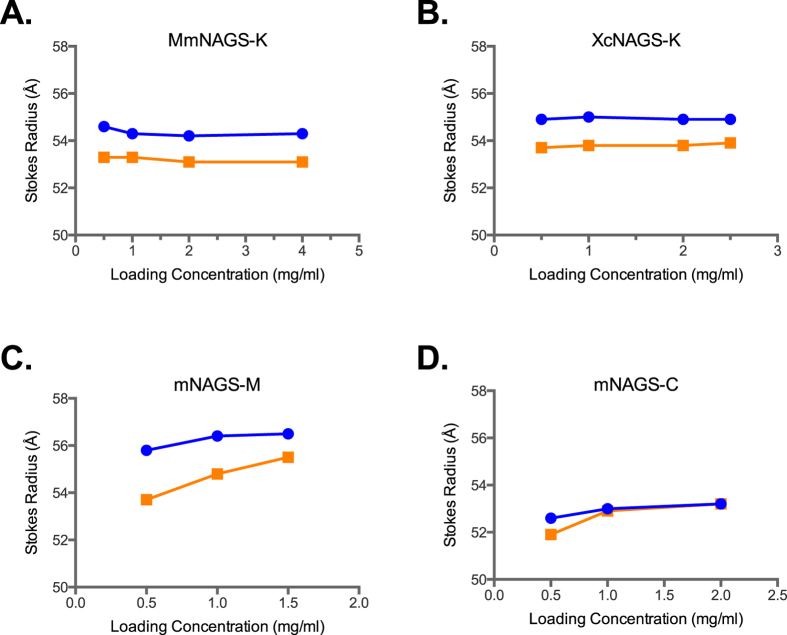
Concentration dependence of Stokes’ radii of MmNAGS-K (**A**), XcNAGS-K (**B**), mNAGS-M (**C**) and mNAGS-C (**D**). Stokes’ radii were determined using analytical gel chromatography (Supplementary Figs S2 and S3) in the presence (orange) and absence (blue) of L-arginine.

The elution volumes and Stokes’ radii of MmNAGS-K and XcNAGS-K were not concentration dependent suggesting that quaternary structure of both proteins does not change in the concentration ranges tested here (Fig. 5A and B). In the presence of 1 mM L-arginine, the Stokes’ radii of both MmNAGS-K and XcNAGS-K decreased (Fig. 5A and B), consistent with a conformational change that results in a smaller hydrodynamic radius. However, this is also consistent with ensembles of MmNAGS-K and XcNAGS-K molecules with different oligomerization states that depend on the presence of L-arginine; the smaller hydrodynamic radius of both proteins could be due to the shift in the equilibrium towards smaller oligomers. When MmNAGS-K and XcNAGS-K were modeled as ellipsoids with 140 Å and 109 Å principal axes^15^,^26^,^33^, the calculated Stokes’ radius of both proteins was 58.8 Å, which is 4 to 6 Å larger than measured values (Fig. 5A and B). This discrepancy is likely due to deviations of the shapes of MmNAGS-K and XcNAGS-K molecules from the idealized ellipsoid.

The mNAGS-M and mNAGS-C had Stokes’ radii of approximately 56 Å and 52 Å, respectively, and the hydrodynamic radii of both proteins decreased in the presence of L-arginine (Fig. 5C and D, Supplementary Fig. S3). The decrease of Stokes’ radii in the presence of L-arginine was concentration dependent, although the magnitude of the change was small (Fig. 5C and D, Supplementary Fig. S3). This suggests that both mNAGS-M and mNAGS-C could be ensembles of molecules with different oligomerization states that exchange rapidly and that binding of L-arginine shifts their equilibrium towards smaller molecules. Alternatively, binding of L-arginine could trigger a conformational change of both mNAGS-M and mNAGS-C that either results in smaller hydrodynamic radius, or in the interactions with the column and a larger elution volume that gives the appearance of smaller hydrodynamic radius.

The apparent molecular weights of mNAGS-M and mNAGS-C were approximately 300 and 240 kDa, respectively, based on their elution volumes. These molecular weights agree most closely with a pentameric oligomerization state based on the calculated molecular weights of 54.7 and 51.0 kDa for the respective mNAGS-M and mNAGS-C monomers. This is unlikely based on the homology model of mammalian NAGS^15^ and different from the primarily tetrameric state of mNAGS-C and human NAGS-C in the absence of L-arginine, which was observed using different analytical gel chromatography conditions^34^. These data and the observation that the hydrodynamic radii of both mNAGS-M and mNAGS-C appear to be concentration dependent in the presence of L-arginine suggest that both proteins may be ensembles of tetramers and larger oligomers and that their equilibrium shifts toward the tetrameric state in the presence of L-arginine.

### Tetramer-Octamer Equilibrium of XcNAGS-K

**Figure 6:**
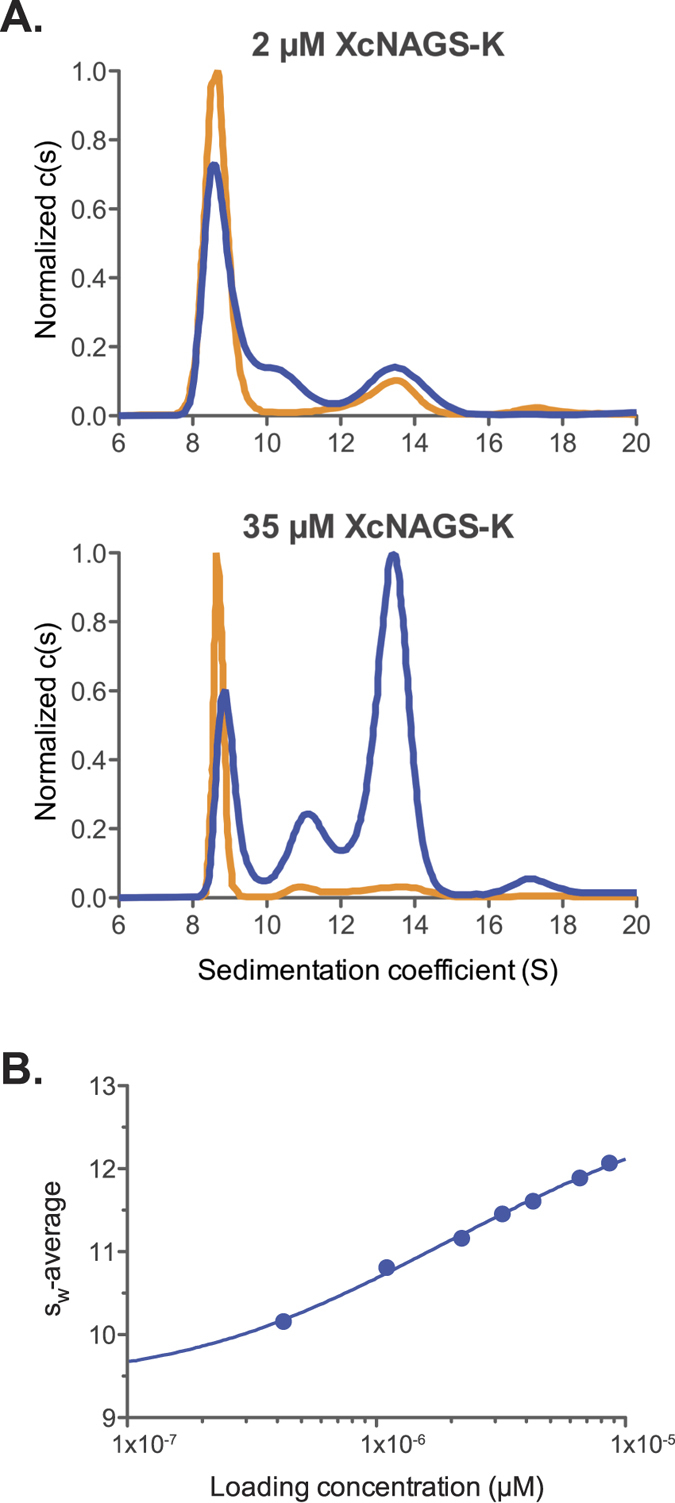
Analytical ultracentrifugation of the XcNAGS-K. (**A**) Distribution of sedimentation coefficients c(s) in the absence (blue) and presence (orange) of L-arginine at two different protein concentrations. Molar concentrations refer to the concentration of XcNAGS-K monomer. (**B**) The S_w_ isotherm for XcNAGS-K in the absence of arginine showing the concentration dependence of the weight-average sedimentation coefficient in the c(s) distributions integrated between 7 and 16 S (solid circles) in overlay with the best-fit curve describing a tetramer-octamer association model.

The discrepancies between calculated and observed molecular weights of MmNAGS-K, XcNAGS-K and the two mouse NAGS variants suggest that, in solution, these proteins may be ensembles of molecules with different oligomerization states. Analytical ultracentrifugation can be used to test this hypothesis. Unfortunately, neither mNAGS-M nor mNAGS-C are suitable for analytical ultracentrifugation because they require high concentration of glycerol, salt and imidazole as well as acetone to prevent aggregation^6^,^21^. When equilibrium analytical ultracentrifugation was used to determine the oligomerization states of MmNAGS-K and XcNAGS-K, the behavior of both proteins deviated from ideality. Therefore, we measured sedimentation velocity to identify whether XcNAGS-K and MmNAGS-K proteins are present in solution in the tetrameric state or as a mixture of oligomers, and to assess the potential effects of L-arginine on oligomer size. We were able to determine the effects of L-arginine only on the oligomerization state of XcNAGS-K because MmNAGS-K aggregated under the conditions used for analytical ultracentrifugation. The sedimentation velocity profiles obtained for XcNAGS-K without L-arginine and in the presence of 1 mM L-arginine look qualitatively similar (Supplementary Figs S4 and S5); at least two distinct boundaries can be discerned from the sedimentation gradients indicating the presence of different sized species. An initial analysis of the velocity profiles resulted in a sedimentation coefficient distribution that shows two to three peaks with sedimentation rates ranging between 8 S and 15 S and a trace amount of material sedimenting beyond 16 S (Fig. 6A). The third peak in the c(s) distribution at ~11 S is likely an artifact of the analysis representing some time-averaged pseudo-species arising from the relative rates of dissociation to sedimentation. Integration of the distribution in this range gives a weight-average sedimentation coefficient s_w_, which increases in a concentration-dependent manner but which was consistently lower in the presence of 1 mM L-arginine at both Xc-NAGS-K loading concentrations (Fig. 6A). Additionally, while the peak positions in the distribution remained relatively fixed, the area under the peaks increased with increased loading concentration. These observations are consistent with a self-associating system whereby dissociation occurs on a time-scale that is intermediate-to-slow relative to the sedimentation process^35^. Therefore, XcNAGS-K does appear to exist in solution as a reversibly-interacting mixture of oligomers; the equilibrium between oligomers of different sizes is altered toward smaller oligomers in the presence of 1 mM L-arginine.

To identify which oligomeric states are represented by the peaks in the distribution of sedimentation coefficients and to gain insight into the association model, we calculated theoretical values of sedimentation coefficients for both tetrameric and octameric complexes and compared them to the weight-average s_w_ of the measured boundaries. A tetrameric XcNAGS-K protein with a reasonably globular overall shape asymmetry (f/fo = 1.25) is predicted to sediment at 9.2 S while the octamer is predicted to sediment at approximately 14.7 S; both calculated values are within the range of observed sedimentation coefficients (Fig. 6A). Deviations of actual overall shape from this globular assumption can lead to differences in the measured sedimentation coefficient from the predicted values. Additionally, the peak position of the reaction boundary can also vary depending on the kinetics of the dissociation process and loading concentration^35^. The correlation of the calculated and measured sedimentation rates and the simplicity of the model suggest an equilibrium between XcNAGS-K tetramers and octamers.

To test the validity of this assembly mechanism, an s_w_ isotherm was constructed showing the concentration dependence of the s_w_-average and was fit using a tetramer-octamer equilibrium association model (Fig. 6B). The best-fit K_d_ from this analysis with 68% confidence interval was 2.9 μM [1.4–5.4] for Xc-NAGS-K in the absence of arginine, where the numbers in bracket represent the errors at a 65% confidence level. An inherent difficulty in fitting s_w_ isotherms for self-associating systems is the uncertainty in the sedimentation coefficient of the oligomers, which translate into errors in the K_d_ estimates. The best-fit values for sedimentation coefficient of the tetramer and octamer were 9.9 S [9.3–10.3] and 14.0 S [13.7–14.6], respectively. These values are qualitatively consistent with the two major peaks in the c(s) distribution (Fig. 6A) and correspond to a frictional ratio of 1.17 for the tetramer and 1.31 for the octamer. Analysis of the s_w_ isotherm for Xc-NAGS-K in the presence of 1 mM L-arginine was ill-conditioned due to the diminished octamer population and uncertainty in s_w_ for the octamer resulting in the best fit K_d_ of 37 μM [1.2–150].

To further confirm the validity of the association mechanism and to take advantage of the rich information contained within the shapes of the sedimentation boundaries, several datasets were globally fit using a tetramer-octamer equilibrium association model with explicit Lamm equation solutions^36^. Supplementary Fig. S2 shows the results of this global analysis for XcNAGS-K in the absence of L-arginine. The best-fit K_d_ was 2.6 μM [2.2–3.1], which is in good agreement with the value obtained from the s_w_-isotherm analysis. Interestingly, the elution volume of XcNAGS-K (Supplementary Fig. S5) corresponding to 277 kDa is exactly what one would expect for a tetramer-octamer equilibrium with a K_d_ of 2.6 μM. The best-fit values for sedimentation coefficients of the tetramer and octamer were 8.95 S and 13.7 S, respectively, which correspond to reasonably globular shape asymmetry factors of 1.29 and 1.34. The best-fit value for the kinetic dissociation constant (log_10_k_off_) was −3.6, which corresponds to a half-life for the octamer of roughly 45 minutes.

### Effect of L-Arginine on NAGS from Different Organisms

**Figure 7:**
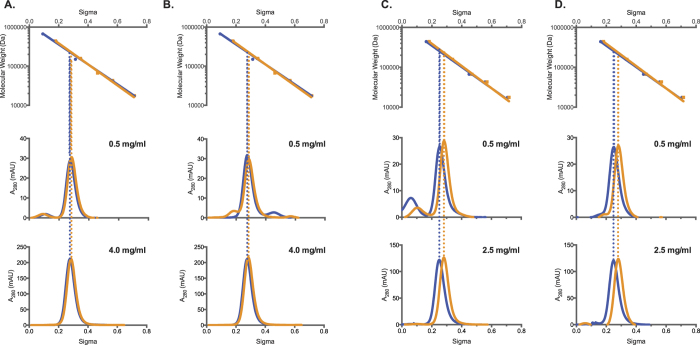
Analytical gel chromatography of MmNAGS-K and XcNAGS-K. (**A)** and (**C**) MmNAGS-K and XcNAGS-K that were allowed to equilibrate overnight at indicated protein concentrations with and without L-arginine. (**B** and **D**) MmNAGS-K and XcNAGS-K were diluted immediately before loading onto column and did not equilibrate with L-arginine prior to loading onto column. The top panels show a semi-logarithmic plot of molecular mass vs. elution volume of size and molecular weight standard proteins. Lower panels show absorption at 280 nm as a function of elution volume and protein concentration. Blue - elution profiles in the absence of L-arginine. Orange – elution profiles in the presence of 1 mM L-arginine.

**Figure 8:**
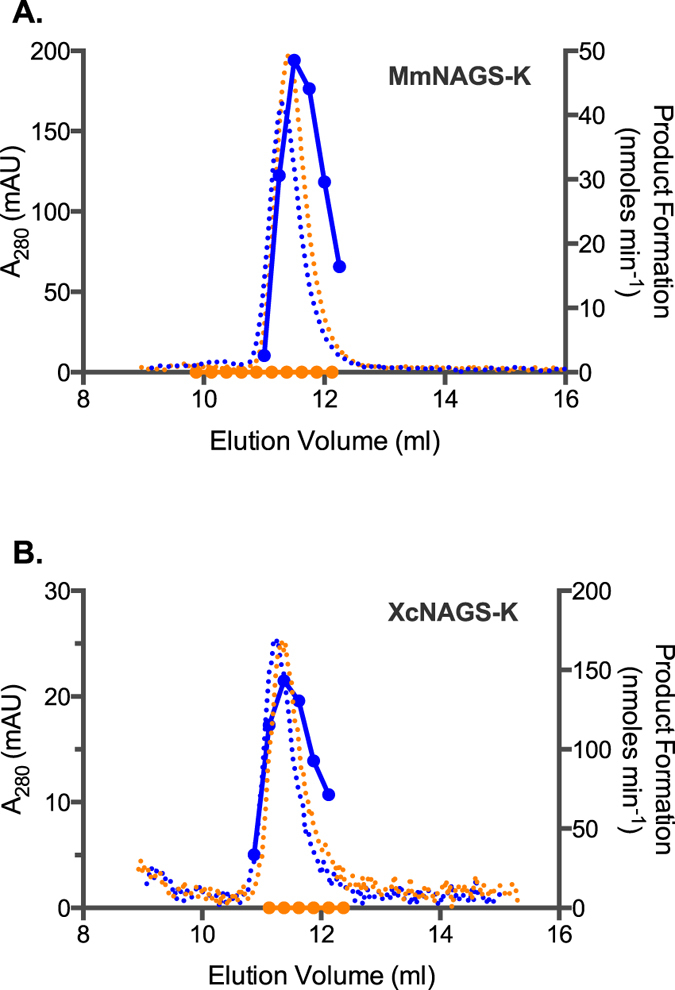
Elution profiles of MmNAGS-K (**A**) and XcNAGS-K (**B**). Absorption at 280 nm (dotted lines) and rate of product formation (solid lines) were measured as functions of elution volume in the absence (blue) or presence (orange) of L-arginine.

Analytical ultracentrifugation of the XcNAGS-K revealed that equilibrium between XcNAGS-K tetramers and octamers depends on the presence and binding of L-arginine and that the rate of exchange between two oligomerization states is moderate to slow. Because MmNAGS-K, mNAGS-M and mNAGS-C were not amenable to analytical ultracentrifugation, we used analytical gel chromatography to examine the effect of L-arginine on their partition coefficients (σ). Behavior of all four proteins was examined at two concentrations, 0.5 mg/ml and the highest concentration attainable for each protein; at each concentration MmNAGS-K, XcNAGS-K, mNAGS-C and mNAGS-M were allowed to equilibrate overnight either with or without 1 mM L-arginine. Proteins that were diluted to 0.5 mg/ml immediately before loading onto column and proteins that were not equilibrated with L-arginine prior to loading onto column were used as controls. MmNAGS-K had the same partition coefficient regardless of the equilibration time prior to loading onto column; additionally, the partition coefficient of MmNAGS-K was not concentration dependent in the presence or absence of 1 mM L-arginine (Fig. 7A and B). The MmNAGS-K partition coefficient was 0.27 in the absence of L-arginine and 0.28 with 1 mM L-arginine. This small change in partition coefficient is consistent with a conformation change that results in a smaller hydrodynamic radius for MmNAGS-K. The partition coefficient of XcNAGS-K in the absence of L-arginine was 0.25 under all experimental conditions and it increased to 0.28 in the presence of 1 mM L-arginine (Fig. 7C and D). The XcNAGS-K partition coefficient was not concentration dependent in the range of concentrations that we could test. The increased XcNAGS-K partition coefficient in the presence of L-arginine is consistent with results of the analytical ultracentrifugation and a shift of the tetramer-octamer equilibrium toward a smaller oligomerization state. Both MmNAGS-K and XcNAGS-K remained active after analytical gel chromatography; elution profiles of enzymatic activity and UV-light absorption were similar for both proteins (Fig. 8). The elution peaks of MmNAGS-K and XcNAGS-K enzymatic activities are broader and trailing compared with UV absorbance (Fig. 8) suggesting that the specific activity of tetrameric forms of both enzymes are higher than the specific activities of the octamers. As expected both proteins were completely inhibited by L-arginine (Fig. 8).

**Figure 9:**
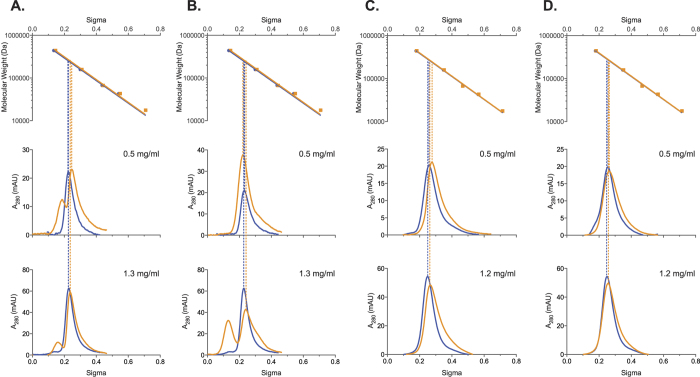
Analytical gel chromatography of mNAGS-C and mNAGS-M. (**A** and **C**) mNAGS-C and mNAGS-M that were allowed to equilibrate overnight at indicated protein concentrations with and without L-arginine. (**B** and **D**) mNAGS-C and mNAGS-M were diluted immediately before loading onto column and did not equilibrate with L-arginine prior to loading onto column. The top panels show a semi-logarithmic plot of molecular mass vs. elution volume of size and molecular weight standard proteins. Lower panels show absorption at 280 nm as a function of elution volume and protein concentration. Blue - elution profiles in the absence of L-arginine. Orange – elution profiles in the presence of 1 mM L-arginine.

**Figure 10:**
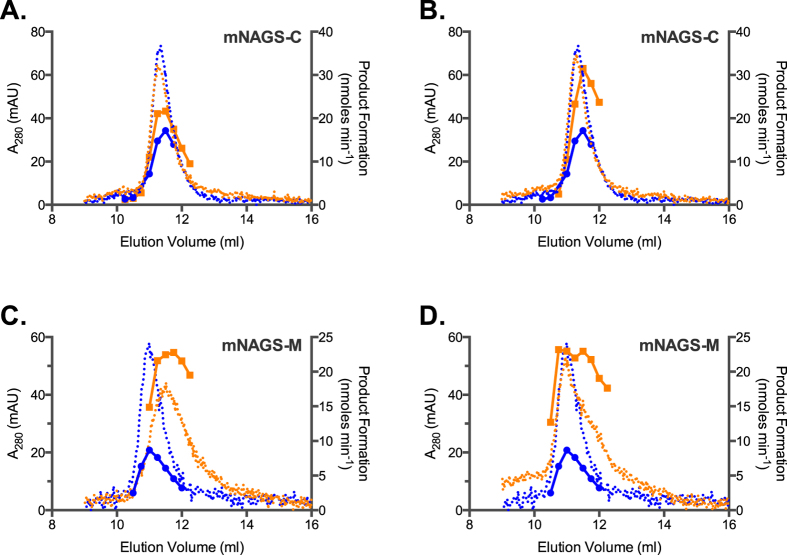
Elution profiles of mNAGS-C and and mNAGS-M. (**A** and **C**) mNAGS-C and mNAGS-M that were allowed to equilibrate overnight with and without L-arginine. (**B** and **D**) mNAGS-C and mNAGS-M did not equilibrate with L-arginine prior to loading onto column. Absorption at 280 nm (dotted lines) and rate of product formation (solid lines) were measured as functions of elution volume in the absence (blue) or presence (orange) of L-arginine.

The mNAGS-C, which lacks a variable segment and resembles MmNAGS-K and XcNAGS-K more than mNAGS-M, eluted as a single peak and had the same partition coefficient of 0.23 at both concentrations that were tested in the absence of L-arginine (Fig. 9A and B). When mNAGS-C was allowed to equilibrate overnight with 1 mM L-arginine, the protein eluted as two overlapping peaks (Fig. 9A). The partition coefficients of the smaller mNAGS-C species were 0.25 and 0.24 for the 0.5 and 1.3 mg/ml loading concentrations, respectively (Fig. 9A). The peaks corresponding to the larger mNAGS-C species are likely to be products of protein aggregation because they were not prominent in other mNAGS-C elution profiles (Fig. S3, 9A, 9B and 10). In control experiments, mNAGS-C, diluted to 0.5 mg/ml, eluted as a single peak in the absence and presence of L-arginine, while more concentrated mNAGS-C (1.3 mg/ml) eluted as a single peak in the absence of L-arginine and as two peaks in the presence of 1 mM L-arginine (Fig. 9B). The partition coefficient of mNAGS-C in the absence of L-arginine was 0.23 at both loading concentrations; in the presence of 1 mM L-arginine partition coefficients changed to 0.22 and 0.24 when loading concentrations of mNAGS-C were 0.5 and 1.3 mg/ml, respectively (Fig. 9B). The peak corresponding to larger mNAGS-C species is likely an aggregation product. The mNAGS-M, which has the variable segment at its N-terminus, eluted as a single peak at all conditions that were tested (Fig. 9C and D). When mNAGS-M was diluted to 0.5 and 1.2 mg/ml and allowed to equilibrate overnight in the absence and presence of 1 mM L-arginine the partition coefficients increased from 0.26 to 0.27 and from 0.25 to 0.27, respectively (Fig. 9C). The behavior of mNAGS-M was similar when it was not allowed to equilibrate overnight in the presence of 1 mM L-arginine (Fig. 9D). The increase in mNAGS-M partition coefficient in the presence of L-arginine is consistent with a conformation change that either results in smaller Stokes’ radius or leads to interaction of mNAGS-M with the column and increases its retention time. Both mNAGS-C and mNAGS-M remained active after analytical gel chromatography. Elution profiles of mNAGS-C and mNAGS-M enzymatic activities resembled their UV-absorption elution profiles both in the presence and absence of L-arginine (Fig. 10).

The mNAGS-C and MmNAGS-K had similar behavior in analytical gel chromatography; partition coefficients of both proteins changed slightly in the presence of L-arginine suggesting that mNAGS-C may be a candidate for structural studies. However, mNAGS-C may not be a physiological form of NAGS because it lacks variable segment and screen of mature forms of mammalian NAGS for proteins that behave like MmNAGS-K could be useful for selecting NAGS suitable for determination of three-dimensional structure.

## Concluding Remarks

Examination of biophysical properties of NAGS proteins from different organisms in this study and earlier studies of the zebrafish, rat and *E. coli* NAGS^9^,^11^,^13^,^25^ revealed flexibility of the NAGS quaternary structures. This property of NAGS is not unique; there are many examples of homologous protein with different oligomerization states^37^,^38^,^39^. Different oligomerization states of some homologous proteins arose due to insertions and deletions of amino acids^37^,^38^ but this mechanism is not responsible for the differences in NAGS quaternary structure based on the alignment of NAGS protein sequences (Supplementary Fig S1) and inspection of available three-dimensional structures. Structural differences between unliganded and L-arginine-bound mammalian NAGS may reveal whether and how L-arginine regulates ureagenesis through modulating enzymatic activity of NAGS.

## Methods

### Protein Purification

NgNAGS, mNAGS-M and mNAGS-C, XcNAGS-K, and MmNAGS-K expression constructs have been described previously^6^,^12^,^16^,^21^. NAGS proteins were purified using nickel affinity chromatography as described previously^6^,^12^. The quality of purification was assessed using SDS-PAGE and enzymatic activity measurements^40^. Enzymes were stored at 4 °C, and catalytic activity was used to determine their stability in storage.

For sedimentation velocity measurements, recombinant XcNAGS was purified using nickel affinity chromatography^12^ followed by size exclusion chromatography. For size exclusion chromatography, purified XcNAGS protein was eluted on a Hiload 16/60 Superdex 200 column (Amersham) at a constant flow rate of 1.0 ml/min in a buffer containing 50 mM Tris pH 8.0, 150 mM KCl, 1 mM Tris[2-carboxyethyl]phosphine (TCEP) using a Pharmacia Acta FPLC system. The protein was concentrated on a Centriprep centrifugal filter device (Milllipore) to get a protein concentration of approximately 2 mg/ml and dialyzed into buffer (10 mM Tris, 50 mM KCl and 1 mM TCEP, pH 8.0) with and without 1 mM arginine.

### Enzyme Activity Measurements

Specific activities of purified enzymes were measured as described previously^40^. Thermal inactivation of enzyme activity was determined by incubating MmNAGS-K and XcNAGS-K at 20, 30, 35, 40, 45, 50, 55, 60 and 70 °C for 10 min., followed by measurement of enzyme activity using standard assay at 30 °C^40^. Mouse NAGS-M and mNAGS-C were incubated without L-arginine at 20, 30, 35, 40, 45, 50, 55 and 60 °C for 10 min., followed by measurement of enzymatic activity at 30 °C and in the absence of L-arginine; mNAGS-M and mNAGS-C were also incubated with 1 mM L-arginine at 20, 30, 35, 40, 45, 50, 55 and 60 °C for 10 min., followed by measurement of enzymatic activity at 30 °C and in the presence of 1 mM L-arginine. L-arginine was in 10,000-fold excess in the thermal inactivation assays. Product formation by MmNAGS-K, XcNAGS-K, mNAGS-M and mNAGS-C after analytical gel chromatography was determined in 250 μl fractions.

### Thermofluor Analysis

Thermal shift assays were performed in a 96 well plate format using a 7900HT Real-Time PCR System (Applied Biosystems). Protein unfolding was monitored by measuring the change in fluorescence intensity of SYPRO Orange (Invitrogen) while ramping temperature from 4 °C to 99 °C. Wells contained 10 μg of enzyme in 50 mM potassium phosphate pH 7.5, 300 mM KCl, 20% glycerol, 250 mM imidazole, 10 mM β-mercaptoethanol (BME), 0.006% Triton X-100, 1% acetone, 50x SYPRO Orange and either L- or D-arginine at concentrations indicated in the text. D-arginine was used as a negative control because it is chemically identical to L-arginine but was expected to be a non-binding enantiomer. Preliminary experiments ruled out any possibility of temperature dependent interactions between SYPRO Orange and the buffer components.

### Analytical Gel Chromatography

All analytical gel chromatography experiments were carried out at room temperature. A Superdex 200 HR 10/30 column (Amersham) was equilibrated with a buffer that contained 50 mM potassium phosphate pH 7.5, 300 mM KCl, 20% glycerol, 10 mM BME, and 0.006% Triton X-100 with and without 1 mM L-arginine at a constant flow rate of 0.75 ml/min using a Pharmacia Acta FPLC system. The column was calibrated with ferritin (molecular weight 440 kDa and Stokes’ radius 61.0 Å), catalase (232 kDa and 52.2 Å), aldolase (158 kDa and 48.1 Å), bovine serum albumin (67 kDA and 37.0 Å), ovalbumin (43 kDA and 27.6 Å), and myoglobin (17.6 kDA and 17.5 Å). Void and internal volume markers were blue dextran and vitamin B12. Recombinant proteins were diluted to concentrations indicated in the figures and loaded onto the column. The Stokes’ radii and molecular weight of recombinant proteins were determined using semi-log plots of either hydrodynamic radii or molecular weights of standards against their partition coefficients^41^.

In the second set of analytical gel chromatography experiments, recombinant proteins were either diluted to 0.5 mg/ml or left at high concentration with or without 1 mM L-arginine and allowed to equilibrate at 4 °C overnight before loading onto Superdex 200 HR 10/30 column. The column was equilibrated with the same buffer as above; the flow rate was 0.5 ml/min. The partition coefficients were calculated using formula (V_e_-V_o_)/(V_t_-V_o_) where V_e_ is the elution volume of the protein of interest, V_o_ is the void volume and V_t_ is the total column volume.

Protein concentrations were measured using the Bradford assay (Biorad) according to manufacturer’s instructions and using bovine serum albumin as a standard. To ensure that integrity of each recombinant protein was not affected by chromatography, the activity of the recombinant NAGS was measured before loading onto the column and after elution from the column.

### Analytical Ultracentrifugation

Experimental samples of XcNAGS-K were prepared by dilution from a concentrated stock using filtered dialysate (10 mM Tris, 50 mM KCl, 1 mM TCEP ± 1 mM L-Arg) at protein loading concentrations ranging from 2 μM to 40 μM monomer XcNAGS-K. Samples were loaded into centrifugation cells with 12-mm charcoal-filled epon centerpieces and sapphire windows and arranged in an An-50 Ti rotor within the ProteomeLab XL-I analytical ultracentrifuge (Beckman Coulter, Indianapolis, IN) as described before^42^). After equilibration at 20 °C, the samples were accelerated to a rotor speed of 50,000 rpm and the resulting concentration gradients were recorded using the Rayleigh absorbance scanner with a detection wavelength of 280 nm. Data were analyzed using direct modeling of the diffusional broadening of the entire set of sedimentation profiles to obtain c(s), a sedimentation coefficient distribution model with noise deconvolution and maximum entropy regularization, using SEDFIT^43^ where frictional ratio and meniscus position were left as floating parameters and optimized during the fitting process. Protein partial specific volume (0.741 mL/g) and molar extinction coefficient at 280 nm (51,240 M^−1^ cm^−1^) of XcNAGS-K were calculated from amino acid composition using SEDFIT^44^. Buffer density (1.00125 g/mL) was measured using an Anton Paar DMA5000M density meter and viscosity (0.01003 P) was calculated from buffer composition using SENTERP (provided by J. Philo).

The weight-average sedimentation coefficient (s_w_) isotherm was generated by integration of the c(s) distribution in the region between 8 and 16 S, excluding trace amounts of the faster sedimenting aggregates above 16 S. The XcNAGS-K s_w_ data were fit to a tetramer-octamer association model in SEDPHAT^45^ with binding constant (log_10_K_a_) and sedimentation coefficient of the tetramer, s_4_ and octamer, s_8_ left as floating parameters and optimized during the fit.

Global modeling of raw sedimentation velocity data using explicit Lamm Equation solutions with reaction kinetics was performed using the multi-method global analysis software platform SEDPHAT (vs. 10.39)^36^,^45^. Sedimentation velocity data at several loading concentrations of XcNAGS in the absence of L-arginine (35, 26, 17, 12, 9, 4, and 2 μM monomer XcNAGS-K) or in its presence (35, 12, and 2 μM monomer XcNAGS-K) were used in the global analysis. Fitting parameters, the equilibrium association constant (log_10_K_a_), and the sedimentation coefficient of the tetramer (s_4_) and octamer (s_8_) were initialized with values obtained from the s_w_ isotherm analysis and sequentially allowed to optimize during fitting. The octamer-tetramer dissociation rate constant (log_10_k_off_) was initialized at −4 and allowed to float. A non-participating species was included in the fit to account for the trace amounts of aggregate sedimenting at >16 S with an s-value, mass and concentration kept as floating parameters.

## Additional Information

**How to cite this article:** Haskins, N. *et al*. Effect of arginine on oligomerization and stability of N-acetylglutamate synthase. *Sci. Rep.* **6**, 38711; doi: 10.1038/srep38711 (2016).

**Publisher's note:** Springer Nature remains neutral with regard to jurisdictional claims in published maps and institutional affiliations.

## Supplementary Material

Supplementary Figures

## Figures and Tables

**Table 1:** Tm values with and without L- and D-arginine.

Protein	L-Arginine			D-Arginine		
	0 mM	1 mM	10 mM	0 mM	1 mM	10 mM
NgNAGS	52.95 ± 0.04^a^	50.47 ± 0.03	53.00 ± 0.15	53.09 ± 0.10	53.09 ± 0.25	52.60 ± 0.22
MmNAGS-K	39.26 ± 0.24	44.55±0.10	49.63 ± 0.04	39.02 ± 0.15	39.32 ± 0.08	38.73 ± 0.18
XcNAGS-K	57.87 ± 0.21	nd^b^	nd	58.11 ± 0.37	57.92 ± 0.49	57.13 ± 0.46
mNAGS-M	48.60 ± 0.14	50.78 ± 0.04	52.96 ± 0.04	48.84 ± 0.10	49.05 ± 0.09	49.58 ± 0.17
mNAGS-C	48.86 ± 0.17	51.48 ± 0.03	53.64 ± 0.06	48.87 ± 0.25	48.89 ± 0.06	49.42 ± 0.42

^a^Values are means of three measurements and the associated standard deviations.

^b^Not determined.
