# Supplementary material for: Effect of arginine on oligomerization and stability of N-acetylglutamate synthase
Source: Sci Rep. 2016 Dec 9;6:38711. doi: 10.1038/srep38711 (PMC5146650; doi:10.1038/srep38711)
Supplement: Supplementary Figures [file srep38711-s1.pdf]

Supplementary Information for:

Effect of arginine on oligomerization and stability of N-acetylglutamate synthase

Haskins N[1], Mumo A[2], Brown PH[3], Tuchman M[1], Morizono H[1], Caldovic L[1,*]

[1]Center for Genetic Medicine Research; Children's National Medical Center, Washington, DC; [2]American Academy of Otolaryngology – Head and Neck Surgery Foundation, Alexandria VA; [3]National Institute of General Medical Sciences, National Institutes of Health, Bethesda, MD.

*Corresponding Author: Ljubica Caldovic, Ph.D.
Department of Integrative Systems Biology
Center for Genetic Medicine Research
Children's National Medical Center
111 Michigan Avenue NW
Washington DC, 20010
E-mail: lcaldovic@cnmcresearch.org
Phone: (202) 476-5819
Fax: (202) 476-6014

```
MmNAGS-K   NPN--------------------A-------------------------------------------PGVRQTIVQL
XcNAGS-K   SLP--------------------AQP----------------------------------------HKQTRQTIVRL
yNAGK      NG-----------------------------------------------------------------FSATRSTVIQL
mNAGS-M    LST--------------------ARAHA----EDAEGAKGRVQSPAVEEPSWTPLP---TPLESPAPPAGRSLVQRDIQAF
hNAGS-M    LST--------------------AWSQPQPPPEEYAGADDVSQSPVAEEPSWVPSPRPPVPHESPEPPSGRSLVQRDIQAF
zfNAGS     SAAEVNRRMSSSRTAGHGSKTPLWSQ----------QESYNHSSLGERSAW----------------SNRTLIYRDVKAF
                                                                                       :  :

MmNAGS-K   LSHM-RDGKEIREYLHRFSGI---DQERFAVIKVGGAVIQ--DDLPGLASALAFLQTVGLTPVVVHGGGPQLDAALEAAD
XcNAGS-K   LSSX-ASAKEISQYLKRFSQL---DAKRFAVVKVGGAVLR--DDLEALTSSLSFLQEVGLTPIVLHGAGPQLDAELSAAG
yNAGK      LNNI-STKREVEQYLKYFTSV---SQQQFAVIKVGGAIIS--DNLHELASCLAFLYHVGLYPIVLHGTGPQVNGRLEAQG
mNAGS-M    LNQCGASPGEARHWLTQFQTCYHSVDKPFAVMEVDEEVIRCPQAVSRLAFALAFLQRMDMKPLVVLGLPTPTA-P-----
hNAGS-M    LNQCGASPGEARHWLTQFQTCHHSADKPFAVIEVDEEVLKCQQGVSSLAFALAFLQRMDMKPLVVLGLPAPTA-P-----
zfNAGS     LREIGGDPREARYWLTHFQRAG--STPAFAVLEVDPSVFDSHEMVQSLAFGLSFLQRMDMKLVVVMGLPAEITED-----
           *        *   :*  *          ***::*.  ::   : :  *:  *:**  :.:  :*: *  .

MmNAGS-K   IPTERVDGLRVTRDEAIPIIRDTLTQANLALVDAIRDAGGRAAAVPRGVFEADIVD-ADKLGRVGEPRHIHLDLVGSAAR
XcNAGS-K   IEKQTVNGLRVTSPHALAIVRKVFQASNLKLVEALQQNGARATSITGGVFEAEYLN-RDTYGLVGEVKAVNLAPIEASLQ
yNAGK      IEPDYIDGIRITDEHTMAVVRKCFLEQNLKLVTALEQLGVRARPITSGVFTADYLD-KDKYKLVGNIKSVTKEPIEASIK
mNAGS-M    ------SGCL-----SFWEAKAQLAQSCKVLVDELRHNAATAVPFFGGGSVLSAAE-PAPHASYGGIVAVETDLLQWCLE
hNAGS-M    ------SGCL-----SFWEAKAQLAKSCKVLVDALRHNAAAAVPFFGGGSVLRAAE-PAPHASYGGIVSVETDLLQWCLE
zfNAGS     ------DHTRS--ATDSPLARTVMVKHCQALTEALQDNSANVMPFFSSEALLQLQDNPLDGSSSGPSVVVDSALLQWTLD
                 .             :  :      *.  :.. .  . ..  .       :        *    :    :

MmNAGS-K   AGQAAILACLGETPDGTLVNINADVAVRALVHALQPYKVVFLTGTGGLLDE-DGDILSSINLATDFGDLMQADWVNGGMR
XcNAGS-K   AGSIPVITSLGETPSGQILNVNADFAANELVQELQPYKIIFLTGTGGLLDA-EGKLIDSINLSTEYDHLXQQPWINGGXR
yNAGK      AGALPILTSLAETASGQMLNVNADVAAGELARVFEPLKIVYLNEKGGIINGSTGEKISMINLDEEYDDLMKQSWVKYGTK
mNAGS-M    SNSIPILCPIGETAARRSVLLDSLEVTASLAKALQPTKIIFLNNSGGLRNN-SQKILSNVNLPADLDLVTNAEWLSIKER
hNAGS-M    SGSIPILCPIGETAARRSVLLDSLEVTASLAKALRPTKIIFLNNTGGLRDS-SHKVLSNVNLPADLDLVCNAEWVSTKER
zfNAGS     CRVIPLVCPVGRDTTGRSSVLRSIQVTTAISQTLQPLKVIFLNSSGGIRNQ-NHKVLGLVSLPGDLPALSCAEWLNEVEQ
           .   .::  :.. .      : :  ..  : : :.* *:::*. .**: :    . :. :.*  :   :    *:.   :

MmNAGS-K   LKLEEIKRLLDDLPLSSSVSITRPSELARELFTHAGSGTLIRRGERIVATDDKSSLDLGRLD-----NLVKAAFGRPAVE
XcNAGS-K   VKIEQIKDLLDRLPLESSVSITRPADLAKELFTHKGSGTLVRRGERVLRATSWDELDLPRLT-----SLIESSFGRTLVP
yNAGK      LKIREIKELLDYLPRSSSVAIINVQDLQKELFTDSGAGTMIRRGYKLVKRSSIGEFPSADALRKALQRDAGISSGKESVA
mNAGS-M    QQIRLIVDVLSRLPHYSSAVITAASTLLTELFSNKGCGTLFKNAERMLRVRNLDSLDQGRLV-----NLVNASFGKKLRE
hNAGS-M    QQMRLIVDVLSRLPHHSSAVITAASTLLTELFSNKGSGTLFKNAERMLRVRSLDKLDQGRLV-----DLVNASFGKKLRD
zfNAGS     KRIGSIAELLNLLPVESSAVLTSANTLLTELFSHKGKNV-----FALHRYSSLEDIDVDRLL-----ALINKSFEKNLRE
            ::  *  :*. **  **. :     *  ***:. * ..       :    .  .:                :  :

MmNAGS-K   GYWDRL--RVDRAFVTESYRAAAITTR---LDGW--VYLDKFAVLDDARGEGLGRTVWNRLVDYAPQLIWRSRTNNPVNG
XcNAGS-K   DYFSNT--KLLRAYVSENYRAAVILTDEGXLGASALIYLDKFAVLDDAQGEGLGRAVWNVXREETPQLFWRSRHNNQVNI
yNAGK      SYLRYLENSDFVSYADEPLEAVAIVKK---DTNV--PTLDKFVCSDAAWLNNVTDNVFNVLRRDFPALQWVVSENDANIA
mNAGS-M    DYLESLRPRLHSIYVSEGYNAAAILTVEPVLGGT--PYLDKFVVSSSRQGQGSGQMLWECLRRDLQTLFWRSRVTNPINP
hNAGS-M    DYLASLRPRLHSIYVSEGYNAAAILTMEPVLGGT--PYLDKFVVSSSRQGQGSGQMLWECLRRDLQTLFWRSRVTNPINP
zfNAGS     DYIASLEGRLHSVYLSEGYSAAAIITTEPVNSGT--PYLDKFVVSSSKQGQGTGQILWECIRQDFSKLFWRSRTTNRINP
            .*          :  *   *..* .            ****.  .    :.    :::        * *    .:

MmNAGS-K   FYFEECDGAVRRDEWTVFWRGEMGPVEVADVVEKAFALPPTLEA--------------P
XcNAGS-K   FYYAESDGCIKQEKWKVFWYGLENFEQIQHCVAHCATRQPTLL---------------G
yNAGK      WHFDKSQGSYLKGGKVLFWYGIDDINTISELVENFVKSCDTASTLNS--------SASS
mNAGS-M    WYFKHSDGSFSNKQWIFFWFGLADIRDSYELVNHAKGLPDSFCKPAS--------DPGS
hNAGS-M    WYFKHSDGSFSNKQWIFFWFGLADIRDSYELVNHAKGLPDSFHKPAS--------DPGS
zfNAGS     WYFKHCDGSFVNGHWIVFWLGLSDIRESYELVEFAKSHPDSFCSLSTTETKPLQQHHGS
           ::: ..:*.  .    .** *  .     . *        :
```

Figure S1. Multiple sequence and structure alignment of human, mouse and zebrafish NAGS, yeast NAGK (yNAGK), and bifunctional MmNAGS-K and XcNAGS-K. Three-dimensional structures 3S6H, 3S6K and 3ZZI of MmNAGS-K, XcNAGS-K and yNAGSK, respectively, were used in the alignment after manual removal of the polyhistidine affinity tag sequences. Reference sequences of human. mouse and zebrafsih NAGS (accession numbers: NP_694551, NP_665828 and XP_685919, respoctively) were used in the alighnment after manual removal of predicted mitochondrial targeting sequences. Proteins were aligned using Expresso structural alignment algorithm (www.tcoffee.org).

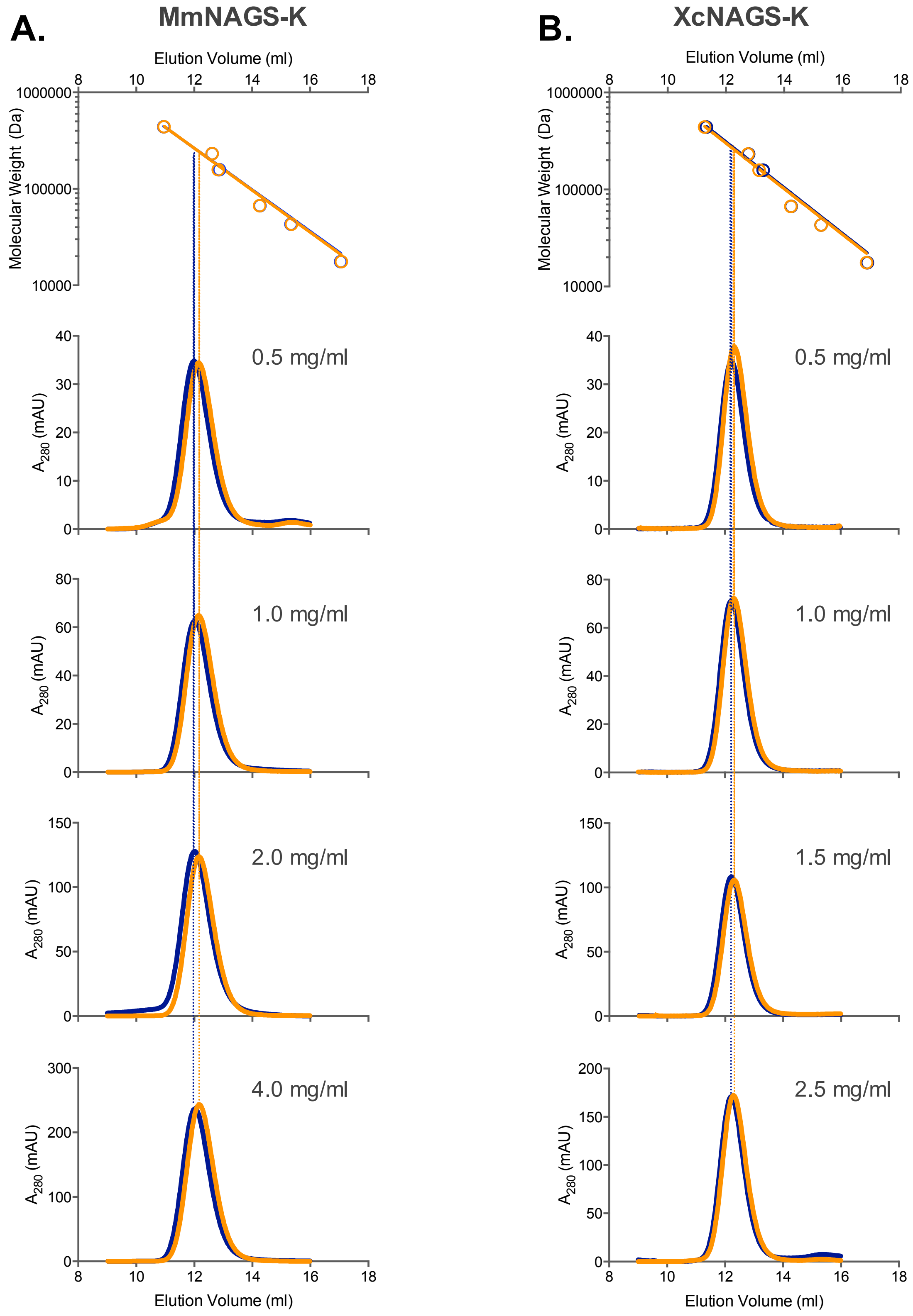

Figure S2. Analytical gel chromatography of MmNAGS-K (A) and XcNAGS-K (B) with and without L-arginine. The top panels show a semi-logarithmic plot of molecular mass vs. elution volume. Open circles correspond to elution volumes of ferritin (440 kDa), catalase (232 kDa), aldolase (158 kDa), bovine serum albumin (66 kDa), ovalbumin (43 kDa), and myoglobin (16 kDa). Lower panels show absorption at 280 nm as a function of elution volume and concentration of each protein loaded on the column. Blue - elution profiles in the absence of L-arginine. Orange – elution profiles in the presence of 1 mM L-arginine.

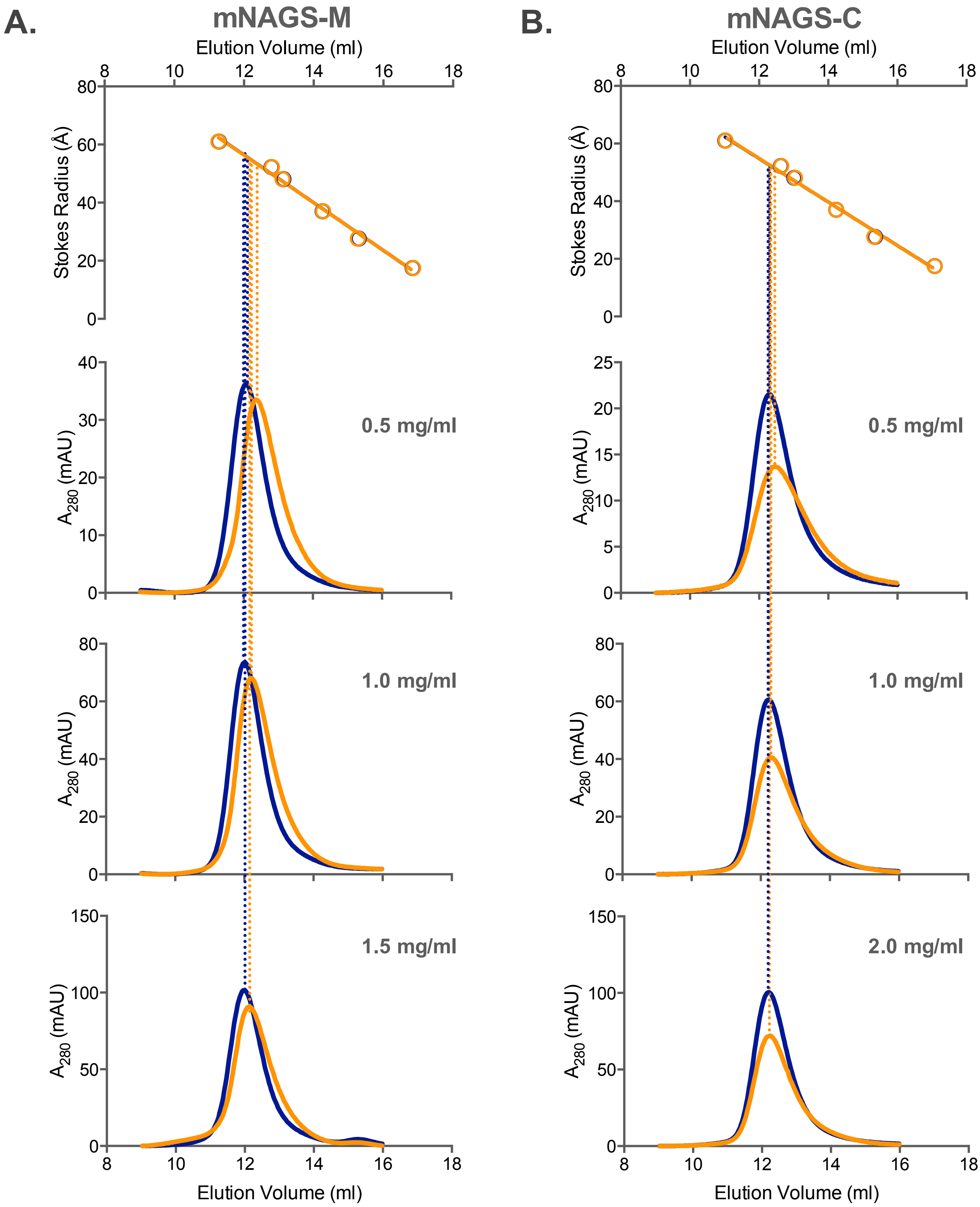

Figure S3. Analytical gel chromatography of mNAGS-M (A) and mNAGS-C (B) with and without L-arginine. The top panels show plots of Stokes' radii vs. elution volume. Open circles correspond to elution volumes of ferritin (61.0 Å), catalase (52.2 Å), aldolase (48.1 Å), bovine serum albumin (37.0 Å), ovalbumin (27.6 Å), and myoglobin (17.5 Å). Lower panels show absorption at 280 nm as a function of elution volume and concentration of each protein loaded on the column. Blue - elution profiles of mNAGS-M and mNAGS-C without arginine. Orange – elution profiles of mNAGS-M and mNAGS-C in the presence of 1 mM arginine.

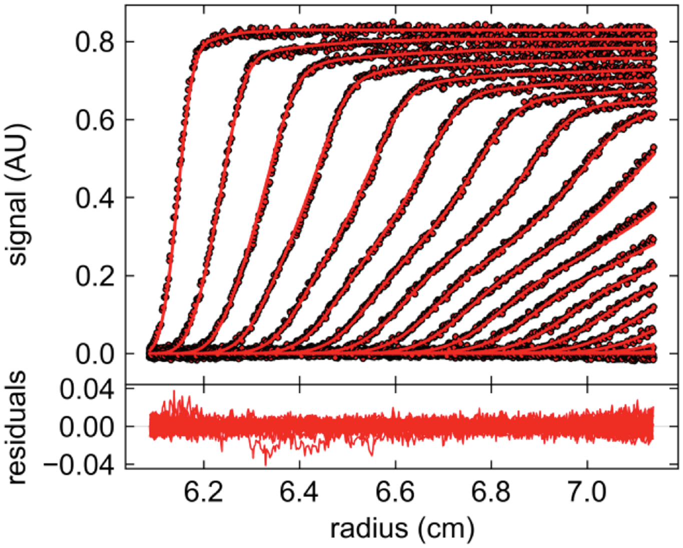

Figure S4. Raw sedimentation velocity profile (black circles) for Xc-NAGS (0.64 mg/mL, which corresponds to 12 μM monomer concentration) in the absence of L-arginine in overlay with the best-fit curves resulting from the c(s) sedimentation coefficient distribution analysis (upper panel).
The residuals from the c(s) analysis with root-mean-square differences of 0.0063 OD (lower panel).

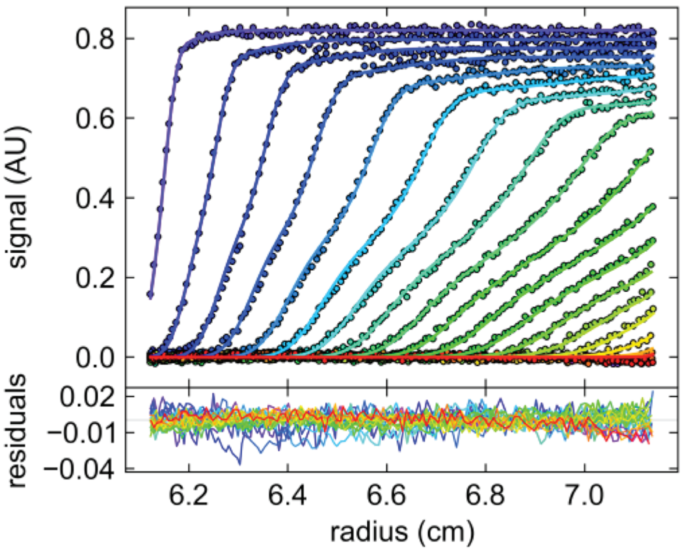

Figure S5. The raw sedimentation velocity profile for XcNAGS (0.64 mg/mL, which corresponds to 12 μM monomer concentration) in presence of 1 mM arginine (circles) overlayed by the best-fit curves resulting from a Global analysis of seven data sets at several loading concentrations (solid lines) fit to a tetramer-octomer association model using explicit Lamm Equation solutions with reaction kinetics. The best fit Kd is 2.6 μM [2.2 – 3.1].
